# Supplementary material for: Development and Validation of an Explainable Radiomics Model to Predict High-Aggressive Prostate Cancer: A Multicenter Radiomics Study Based on Biparametric MRI
Source: Cancers (Basel). 2024 Jan 1;16(1):203. doi: 10.3390/cancers16010203 (PMC10778513; doi:10.3390/cancers16010203)
Supplement: Supplementary file 1 [file cancers-16-00203-s001.zip › cancers-2748445-supplementary.pdf]

Supplementary Section S1 – Reporting Guidelines: from Image acquisition to Data conversion

Area	Topic	Description
**Patient**	Region of interest	Pelvic zone for prostate cancer staging
**Acquisition**	Acquisition protocol	Different acquisition protocols
	Scanner type	Achieva, Philips Medical Systems, at center A.<br><br>Ingenia, Philips Medical Systems, at center B.<br><br>Optimae, GE HealthCare, at center C.<br><br>Magnetom Trio, Siemens Medical Solution, at center D.
	Imaging modality	Magnetic Resonance
	Scanning sequence	At least diffusion weighted (DWI) and T2-weighted (T2w) imaging
	Acquisition type	2D
	Magnetic field strength	1.5T at centers A, B, and C.<br><br>3T at center D
**Reconstruction**	In-plane resolution	Distance between pixels: 0.4687x0.4687mm, 0.6875x0.6875mm, 0.3125x0.3125mm, 0.625x0.625mm, for T2w imaging respectively at centers A, B, C, and D.<br><br>Distance between pixels: 1.875x1.875, 2.2321x2.2321, 0.7813x0.7813mm, 1.3888x1.3888mm, for DWI respectively at centers A, B, C, and D.
	Image slice spacing	3mm for both T2w and DWI imaging at center A, B, C, and D
	Image slice thickness	3mm for both T2w and DWI imaging at center A, B, C, and D
	Diffusion weighted imaging – b value	Low b-value: either 0 or 50* s/mm^2<br><br>High b-value: either 800* or 1000 s/mm^2<br><br>*For patients acquired with more than two b-values, we chose to standardize the ADC map computation by selecting the b-value closest to 0 s/mm^2 and 1000 s/mm^2.
**Data conversion**	ADC computation	ADC= $\ln(S_0/S_1)/(b_1-b_0)$, where S_0 and S_1 are the signal intensity obtained with the b_0 and b_1 b values,

		respectively.
	Other data conversions	-

Table S1 - *Reporting Guidelines: from Image acquisition to Data conversion*

Supplementary section S2 - Reporting Guidelines: from Image pre-processing to features calculation steps

First, each tumor volume was normalized by dividing each voxel by the maximum signal intensity value of the volume and then interpolated to have the same pixel resolution for images coming from different centers (0.5 mm along x and y). Even though the absolute ADC values already have a physical meaning, we were forced to normalize also ADC images to face the fact that acquisition parameters across scanners from different vendors and between models from the same manufacturer can widely affect ADC. Then, the pixel range of the ADC map was set between the 1st and the 95th percentiles and then all other pixels were removed from the mask to eliminate the most hypo and hyperintense pixels of the segmentation considered as possible outliers. Similarly, the T2w image's pixel range was set between the 1st and the 99th percentiles to remove outlier pixels due to possible inaccuracies in the manual segmentation. The 95th and 99th percentiles for the pixel range of ADC and T2w imaging were chosen based on preliminary analyses which guaranteed the removal of most of the non-tumoral pixels present inside the tumor segmentation. Then, all images were discretized using a fixed number of bins (n=32).

Texture features were extracted from the Gray Level Co-occurrence Matrix (GLCM), Gray Level Run Length Matrix (GLRLM), Gray Level Size Zone Matrix (GLSZM), Neighboring Gray Tone Difference Matrix (NGTDM), and Gray Level Dependence Matrix (GLDM). In addition, we computed the tumor volume from the segmented mask and first-order intensity-based features only from the ADC map since T2w acquisitions suffer from high variability between scanners and acquisition protocols.
Features were extracted for each slice of the axial plane and then averaged, and with "*no_weighting*" value for the texture matrix weighting, i.e., to obtain GLCM and GLRLM features from a 2D slice, matrices were computed for the four possible angles, then weighted by weighting factor 1, summed, normalized, and finally features were calculated on the resultant matrix.

**Post-acquisition processing**	Anti-aliasing	-
	Noise suppression	-
	Non-uniformity correction	-
	Intensity normalization	Each tumor volume is normalized by its maximum value
**Segmentation**	Method	Manual segmentation on both T2w and ADC map
	Conversion to mask	NIFTI
**Image Interpolation**	Interpolation method	Linear interpolation
	Voxel dimension	0.5x0.5x3 mm^3
**ROI Interpolation**	Interpolation method	Linear interpolation
	Partially masked	-

	voxel	
**Re-segmentation**	Methods	Between 1st and 99th percentile for T2W and between 1st and 95th percentile for ADC map
**Discretization**	Method	Fixed bin number (32 bin)
**Image transformation**	Image filter	-
**Image biomarker computation**	Biomarker set	Intensity-based statistics (from ADC maps);<br><br>GLCM, GLRLM, GLSZM, NGTDM, GLDM (from both ADC maps and T2w images)
	IBSI compliance	Yes
	Robustness	-
	Software availability	Pyradiomics 3.0.1
**Image biomarker computation - texture parameters**	Texture matrix aggregation	GLCMs and GLRLMs are averaged over slices and directions;<br><br>GLSZM, NGTDM, GLDM are averaged over slices
	Distance weighting	No weighting
	CM symmetry	Symmetric co-occurrence matrices
	CM distance	Infinity norm distance of 1
	SZM linkage distance	Infinity norm distance of 1
	NGTDM distance	1
**List of features**	(from tumor mask)	- ROI volume
	Intensity-based statistics (from ADC only)	- 10th<br>- 90th<br>- Energy<br>- Entropy<br>- Interquartile Range<br>- Kurtosis<br>- Maximum<br>- Mean<br>- Mean Absolute Deviation<br>- Median<br>- Minimum<br>- Range<br>- Robust Mean Absolute Deviation

		- Root Mean Squared<br>- Skewness<br>- Total Energy<br>- Uniformity<br>- Variance
	GLCM (from both T2w and ADC)	- Autocorrelation<br>- Cluster Prominence<br>- Cluster Shade<br>- Cluster Tendency<br>- Contrast<br>- Correlation<br>- Difference Average<br>- Difference Entropy<br>- Difference Variance<br>- Inverse Difference<br>- Inverse Difference Moment<br>- Inverse Difference Moment Normalized<br>- Inverse Difference Normalized<br>- Information Measure of Correlation 1<br>- Information Measure of Correlation 2<br>- Inverse Variance<br>- Joint Average<br>- Joint Energy<br>- Joint Entropy<br>- Maximal Correlation Coefficient<br>- Maximum Probability<br>- Sum Average<br>- Sum Entropy<br>- Sum Squares
	GLRLM (from both T2w and ADC)	- Gray Level Non-Uniformity<br>- Gray Level Non-Uniformity Normalized<br>- Gray Level Variance<br>- High Gray Level Run Emphasis<br>- Long Run Emphasis<br>- Long Run High Gray Level Emphasis<br>- Long Run Low Gray Level Emphasis<br>- Low Gray Level Run Emphasis<br>- Run Entropy<br>- Run Length Non-Uniformity<br>- Run Length Non-Uniformity Normalized<br>- Run Percentage<br>- Run Variance<br>- Short Run Emphasis<br>- Short Run High Gray Level Emphasis<br>- Short Run Low Gray Level Emphasis
	GLSZM (from both T2w and ADC)	- Gray Level Non-Uniformity<br>- Gray Level Non-Uniformity Normalized<br>- Gray Level Variance<br>- High Gray Level Zone Emphasis<br>- Large Area Emphasis<br>- Large Area High Gray Level Emphasis<br>- Large Area Low Gray Level Emphasis

		- Low Gray Level Zone Emphasis<br>- Size Zone Non-Uniformity<br>- Size Zone Non-Uniformity Normalized<br>- Small Area Emphasis<br>- Small Area High Gray Level Emphasis<br>- Small Area Low Gray Level Emphasis<br>- Zone Entropy<br>- Zone Percentage<br>- Zone Variance
	NGTDM (from both T2w and ADC)	- Busyness<br>- Coarseness<br>- Complexity<br>- Contrast<br>- Strength
	GLDM (from both T2w and ADC)	- Dependence Entropy<br>- Dependence Non-Uniformity<br>- Dependence Non-Uniformity Normalized<br>- Dependence Variance<br>- Gray Level Non-Uniformity<br>- Gray Level Variance<br>- High Gray Level Emphasis<br>- Large Dependence Emphasis<br>- Large Dependence High Gray Level Emphasis<br>- Large Dependence Low Gray Level Emphasis<br>- Low Gray Level Emphasis<br>- Small Dependence Emphasis<br>- Small Dependence High Gray Level Emphasis<br>- Small Dependence Low Gray Level Emphasis

Table S2 - *Reporting Guidelines: from Image pre-processing to features calculation steps*

Supplementary Section S3 – Feature selection algorithms and classifiers

Description of the feature selection methods:

1) Minimum redundancy maximum relevance (MRMR), an algorithm that minimizes the redundancy of the feature set while maximizing its relevance. It measures the pairwise mutual information of features and mutual information of each feature and the desired output.
2) Affinity propagation (AP), a clustering algorithm applied to features. Once clusters were obtained, the prototype of each cluster was selected and included in the optimal subset.
3) Features ranking using Chi-squared (Chi2) tests: features with a score higher than T*ChiMax were selected, where ChiMax is the maximum score obtained and T is a tuned parameter chosen by maximizing the area under the ROC (AUC).
4) Mann-Whitney (M-W) U test was performed between each predictor and the desired output. Only significant features were selected (p-value <0.05).
5) Stepwise binomial logistic regressor (SLR), that automatically adds and removes features during the training phase based on the p-value for an F-test of the change in the deviance that results from adding or removing the term.

Regarding the ensemble learner classifiers, an important aspect to highlight is that different types of classifiers are tuned depending on the “method” and “learner” parameters set in the Matlab function fitcensemble. In this study, we set the option “OptimizeHyperparameters” = “all” which automatically optimize all eligible parameters, including five ensemble aggregation methods (Bag, GentleBoost, LogitBoost, AdaBoostM1, and RUSBoost) and three different learners (discriminant, knn, and tree). This means, for example, that this function includes the training of random forest classifiers since when the “Bag” method is evaluated the function uses bagging with random predictor selections at each split by default.

Note also that the *fitcnb* function automatically computes the prior probabilities for each class as the class relative frequencies in the output vector.

For more information about default parameters refer to the function and parameters specified in Table A.3 of MATLAB 2021b.

**Algorithm**	**MATLAB function**	**Set or tuned parameters**
MRMR	*fscmrmr*	---
AP	-	Distance = Euclidean, maximum number of iterations = 200, number of stall iterations to stop = 20, dampening factor = 0.5
Chi2	*fscchi2*	T in the range [0.6,0.9] in step of 0.1
M-W U test	*ranksum*	--
SLR	*stepwiseglm*	Distribution = binomial, model type = linear, upper = ‘linear’
DT	*fitctree*	Tuning of the minimum leaf size<br>(option ‘OptimizeHyperparameters’ = ‘all’ in MATLAB 2021b)
SVM	*fitcsvm*	Tuning of the kernel, kernel scale, and box constrains<br>(option ‘OptimizeHyperparameters’ = ‘all’ in MATLAB 2021b)
EL	*Fitcensemble*	Tuning of method, number of learning cycles, learning rate, and learner<br>(option ‘OptimizeHyperparameters’ = ‘all’ in MATLAB 2021b)
NB	*fitcnb*	Tuning of type of distribution and width<br>(option ‘OptimizeHyperparameters’ = ‘all’ in MATLAB 2021b)
LR	*fitglm*	Distribution = binomial
LASSO LR	*fitclinear*	‘Regularization’ = ‘lasso’ ; ‘learner’ = ‘logistic’

Table S3 - *Feature selection algorithms and classifiers: Matlab function used and set/tuned parameters*. AP = Affinity propagation, Chi2 = chi-squared, DT = decision tree, EL = ensemble learner, LR = logistic regressor, MRMR = Minimum redundancy maximum relevance, M-W U test = Mann-Whitney U test, NB = naïve Bayes, SLR = Stepwise binomial logistic regressor, SVM = support vector machine.

Supplementary Section S4 - Partial Dependence Plot

The partial dependence plot of each feature employed in the model was created to provide a cohort interpretation of the final model. In this type of plot, the feature's marginal effect on the predicted outcome is provided, showing how the average prediction in the dataset changes when the feature is changed. In figure A1, Partial Dependence plots of the ten features employed in the final classifier are reported. Interestingly, in the partial dependence plots of ADC_glrlm_RunLengthNonUniformity, ADC_glrlm_RunLengthNonUniformityNormalized, T2_glcm_ClusterProminence, and T2_glrlm_ShortRunEmphasis, the probability of each class is characterized by a well-separated probability distribution. Specifically, the two plots of ADC_glrlm_RunLengthNonUniformity and T2_glcm_ClusterProminence show a high probability of low-aggressive PCa for low values of the feature, a switching point from which the probability of low-aggressiveness drops to zero and the probability of high-aggressiveness starts increasing (respectively around 0.1 and 0.3 for ADC_glrlm_RunLengthNonUniformity and T2_glcm_ClusterProminence), and then a high probability of high-aggressive PCa for high values of the feature. Vice versa, ADC_glrlm_RunLengthNonUniformityNormalized, and T2_glrlm_ShortRunEmphasis are characterized by a high probability of high-aggressive PCa for low values of the feature and high probability of low-aggressive PCa for high values of the feature, with a switching point of around 0.3 in both cases. On the contrary, the Partial Dependence plots of the remaining six features display two overlapping probability distributions for the two classes. Coherently with the literature for which there is a negative correlation between ADC values and PCa malignancy, the probability of low-aggressive PCa increases with ADC_firstorder_Mean.

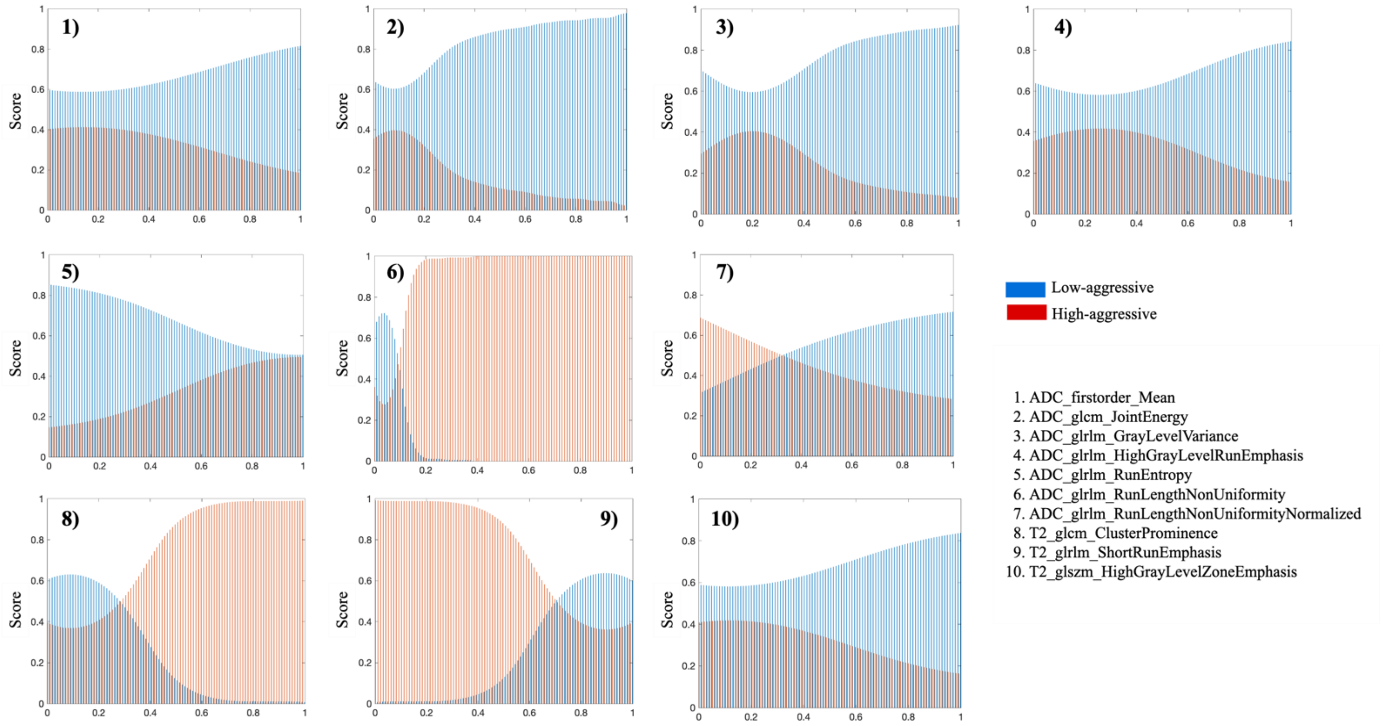

Figure S1 – *Partial Dependence Plots (PDP) of the ten features employed in the final model.*

Supplementary Section S5 - Comparison of feature selection techniques and classifiers

The number of features selected by the MRMR, Chi2, AP, M-W U test, and SGLR in the five folds was 2.4 ± 0.5, 4.3 ± 2.1, 10.8 ± 0.4, 10.6 ± 4.4, and 48.8 ± 7.5, respectively. Regarding the FS techniques, in the CV step the AP achieves the highest value of averaged accuracy (71%) while the M-W U test is the one with the lowest value (50%). The MRMR is the most robust across different classifiers (averaged balanced accuracy from 58% to 65%) while the M-W U test is the less robust (averaged balanced accuracy from 50% to 65%) and, therefore, seems to be more sensitive to the choice of the classifier. Regarding the different tested classifiers, the NB is the model that achieves the highest value of averaged balanced accuracy (71%) while the DT is the one with the lowest value (50%). In addition, the SVM obtains the most robust performances, i.e., the smallest range (averaged balanced accuracy from 61% to 67%) while LR is the one with the most variable performances (averaged balanced accuracy ranging from 52% to 66%) and, therefore, seems to be more sensitive to the choice of the FS method.

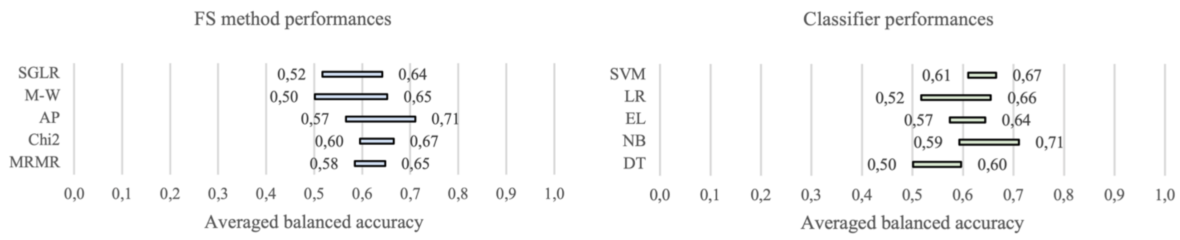

(A) (B)

Figure S2 – *Range of balanced accuracy obtained by the different FS-classifier combinations averaged in the 5 left-out folds and grouped by FS method (A) and classifier (B).* AP = Affinity propagation, Chi2 = chi-squared, DT = decision tree, EL = ensemble learner, LR = binomial logistic regressor, MRMR = Minimum redundancy maximum relevance, M-W = Mann-Whitney U test, NB = naïve Bayes, SGLR = Stepwise binomial logistic regressor, SVM = support vector machine.

		5-fold CV Training performances						5-fold CV Test performances					
		AUC	Bal acc	sens	spec	PPV	NPV	AUC	Bal acc	sens	spec	PPV	NPV
Chi-squared - SVM (polynomial)	mean	0.764	0.724	0.730	0.718	0.767	0.679	0.728	0.665	0.642	0.689	0.730	0.601
	std	0.050	0.057	0.067	0.046	0.035	0.061	0.032	0.094	0.071	0.118	0.074	0.043
Chi-squared - LR	mean	0.734	0.709	0.760	0.659	0.745	0.689	0.687	0.639	0.665	0.613	0.688	0.604
	std	0.012	0.092	0.082	0.102	0.047	0.034	0.071	0.158	0.180	0.137	0.047	0.078
Chi-squared - NB	mean	0.726	0.670	0.691	0.649	0.717	0.625	0.702	0.655	0.684	0.626	0.707	0.631
	std	0.030	0.068	0.063	0.073	0.025	0.024	0.075	0.173	0.197	0.148	0.064	0.093
AP - SVM	mean	0.762	0.707	0.719	0.695	0.751	0.663	0.680	0.643	0.673	0.614	0.699	0.617
	std	0.014	0.060	0.066	0.053	0.024	0.033	0.037	0.171	0.159	0.184	0.108	0.161
AP - NB	mean	**0.772**	**0.714**	**0.694**	**0.734**	**0.769**	**0.656**	**0.751**	**0.711**	**0.694**	**0.728**	**0.788**	**0.669**
	std	0.045	0.051	0.060	0.043	0.014	0.037	0.017	0.169	0.148	0.190	0.122	0.081
M-W U test – SVM (polynomial)	mean	0.731	0.675	0.658	0.692	0.732	0.616	0.660	0.652	0.643	0.662	0.714	0.610
	std	0.057	0.091	0.089	0.093	0.066	0.074	0.052	0.213	0.215	0.211	0.100	0.107
LASSO regression	Mean	0.725	0.691	0.661	0.720	0.751	0.623	0.646	0.631	0.577	0.686	0.702	0.563
	Std	0.019	0.029	0.07	0.38	0.016	0.047	0.055	0.086	0.116	0.100	0.072	0.116

Table S4 - *5-fold cross-validation performances of the feature selection-classifier combinations that achieved performances higher than 60% (in all metrics) in the left-out folds of the cross-validation. Best combination in bold*. AP = affinity propagation, AUC, area under the ROC, Bal acc = balanced accuracy, LR = binomial logistic regressor, M-W U test = Mann-Whitney U test, NB = naïve Bayes, NPV = negative predictive value, PPV = positive predictive value, sens = sensitivity, spec = specificity, SVM = support vector machine.

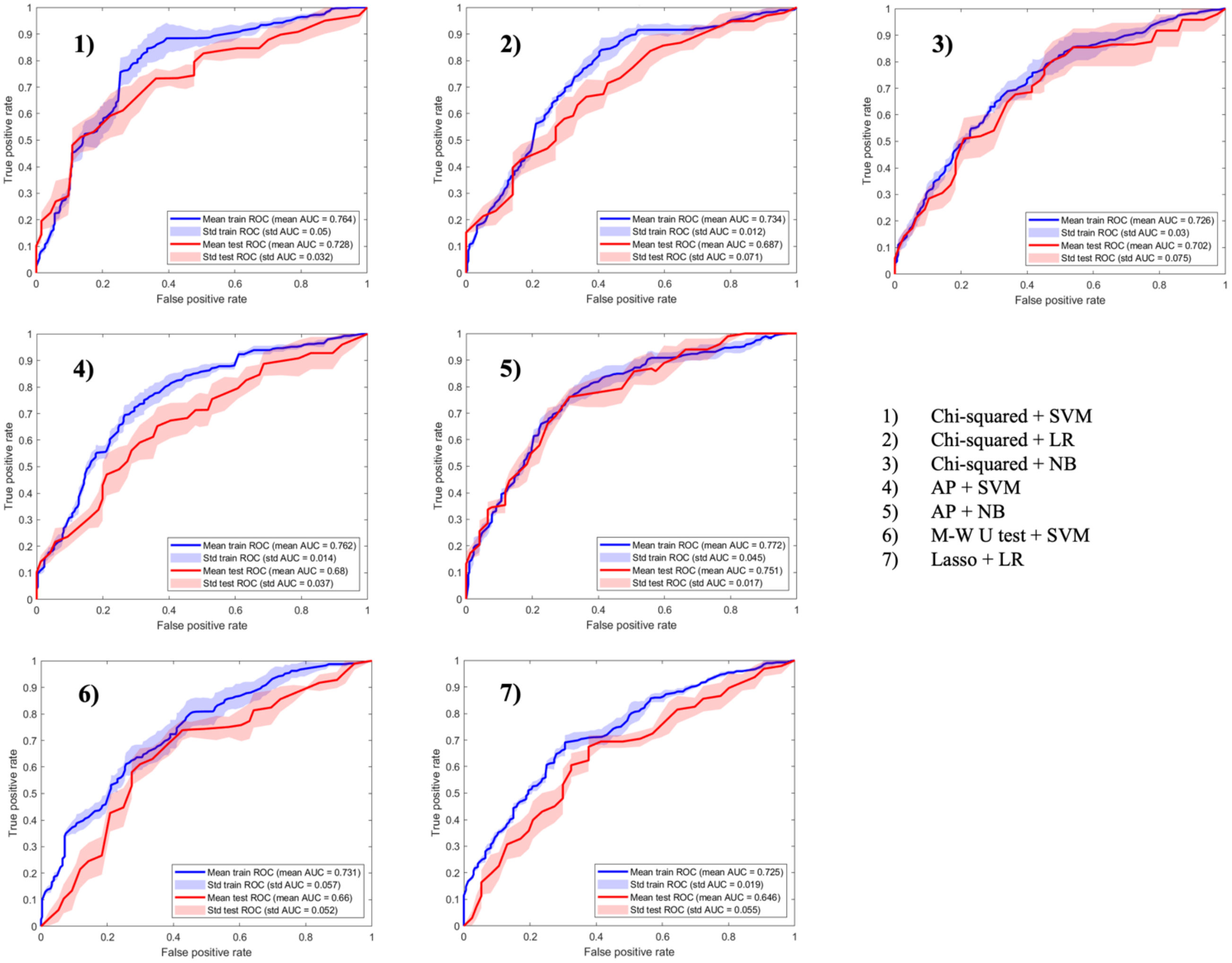

Figure S3. – *Receiver Operating Characteristic (ROC) curve of the feature selection-classifier combinations that achieved performances higher than 60% (in all metrics) in the left-out folds of the cross-validation. Specifically, the ROC curves are reported in terms of mean (line) and standard deviation (smoothed area) values of the train (blue) and test (red) folds of the 5-fold cross-validation.* AP = affinity propagation, AUC, area under the ROC, LR = binomial logistic regressor, M-W U test = Mann-Whitney U test, NB = naïve Bayes, SVM = support vector machine.

Observing all the combinations that achieved performances higher than 60% in the CV phase, no combinations were excluded for overfitting as in no case there was a decrease in performances between training and test sets higher than 30%. The best-performing combination is the AP-NB which achieved an AUC, mean balanced accuracy, sensitivity, specificity, PPV, and NPV of 0.772, 71.4%, 69.4%, 73.4%, 76.9%, and 65.6%, respectively on the training set, and of 0.751, 71.1%, 69.4%, 72.8%, 78.8%, and 66.9%, respectively on the test set.

Observing the differences in the mean performances of the 5-fold CV between the train and test sets (Table 1), the Chi2-SVM and AP-SVM are the two combinations that have the highest decrease in performances in the left-out folds, therefore it could be hypothesized that they are less generalizable than the other combinations. The model that obtained the lowest difference in the mean performances of the 5-fold CV between the train and test is the AP-NB, confirming it as the best FS-classifier combination.

	∂ (test-train)					
	**AUC**	**balanc acc**	**sens**	**spec**	**PPV**	**NPV**
**Chi-squared – SVM (polynomial)**	-0,036 (-4,7%)	-0,059 (-8,1%)	-0,088 (-12,1%)	-0,029 (-4%)	-0,037 (-4,8%)	-0,078 (-11,5%)
**Chi-squared – LR**	-0,047 (-6,4%)	-0,07 (-9,9%)	-0,095 (-12,5%)	-0,046 (-7%)	-0,057 (-7,7%)	-0,085 (-12,3%)
**Chi-squared – NB**	-0,024 (-3,3%)	-0,015 (-2,2%)	-0,007 (-1%)	-0,023 (-3,5%)	-0,01 (-1,4%)	0,006 (1%)
**AP – SVM (polynomial)**	-0,082 (-10,8%)	-0,064 (-9,1%)	-0,046 (-6,4%)	-0,081 (-11,7%)	-0,052 (-6,9%)	-0,046 (-6,9%)
**AP - NB**	-0,021 (-2,7%)	-0,003 (-0,4%)	0 (0%)	-0,006 (-0,8%)	0,019 (2,5%)	0,013 (2%)
**M-W U test – SVM (polynomial)**	-0,071 (-9,7%)	-0,023 (-3,4%)	-0,015 (-2,3%)	-0,03 (-4,3%)	-0,018 (-2,5%)	-0,006 (-1%)
**LASSO – LR**	-0,079 (-10,9%)	-0,06 (-8,7%)	-0,084 (-12,7%)	-0,034 (-4,7%)	-0,049 (-6,5%)	-0,06 (-9,6%)

Highest Value

Lowest Value

Table S5 - *Mean differences between test and training performances in the 5-fold cross-validation. Columns are color-coded from the lowest value of the column in red, to the highest value of the column in green. In parenthesis, the difference percentage compare to the training performances*. AP = affinity propagation, balanc acc = balanced accuracy, LR = binomial logistic regressor, M-W U test = Mann-Whitney U test, NB = naïve Bayes, NPV = negative predictive value, PPV = positive predictive value, sens = sensitivity, spec = specificity, SVM = support vector machine.

Supplementary Section S6 – List of features selected by the affinity propagation algorithm and used to train the final classifier

Imaging type	Selected features
ADC map (7)	First-order - Mean<br>GLCM – Joint Energy<br>GLRLM – Gray Level Variance,<br>GLRLM – High Gray Level Run Emphasis,<br>GLRLM – Run Entropy,<br>GLRLM – Run Length Non-Uniformity<br>GLRLM – Run Length Non-Uniformity Normalized
T2w imaging (3)	GLCM- Cluster Prominence,<br>GLRLM - Short Run Emphasis,<br>GLSZM - High Gray Level Zone Emphasis

Table S6 - *List of features selected by the affinity propagation algorithm and used to train the final classifier*. ADC = apparent diffusion coefficient, GLCM = grey-level cooccurrence matrix, GLRLM = grey-level run length matrix, GLSZM, grey-level size zone matrix, T2w = T2-weighted.

Supplementary Section S7 – Standards for Reporting of Diagnostic Accuracy Studies (STARD) diagram

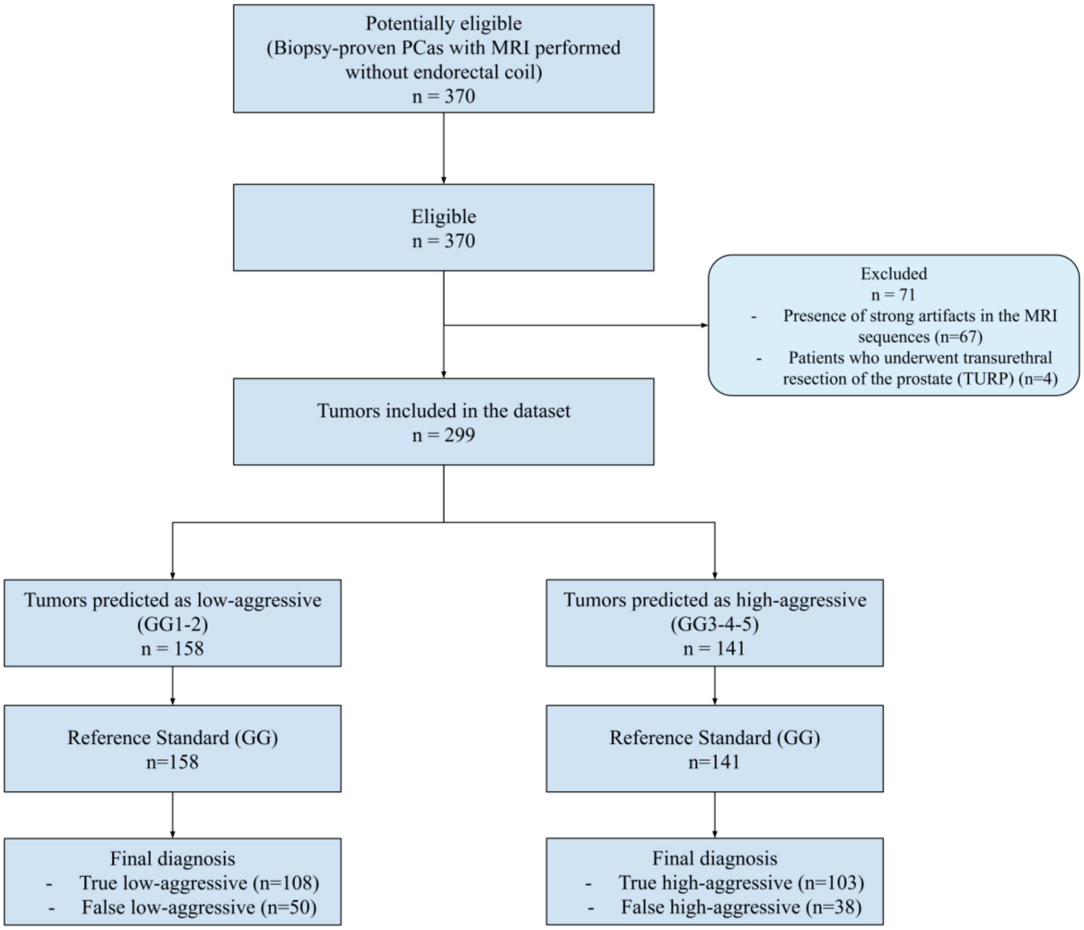

Figure S4 – Standards for Reporting of Diagnostic Accuracy Studies (STARD) diagram. GG=grade group, MRI=magnetic resonance imaging, TURP=transurethral resection of the prostate.

Supplementary Section S8 - Radiomics Quality Score (RQS)

ITEM	Point	Comment
Image protocol quality - well-documented image protocols (for example, contrast, slice thickness, energy, etc.) and/or usage of public image protocols allow reproducibility/replicability	+1 (protocols well documented)	This was a retrospective study therefore it was not possible to use public available protocols.
Multiple segmentations - possible actions are: segmentation by different physicians/algorithms/software, perturbing segmentations by (random) noise, segmentation at different breathing cycles. Analyse feature robustness to segmentation variabilities	N. A.	Manual segmentation is a very time-consuming task and it is not feasible to have more than one radiologist segmenting the whole dataset.
Phantom study on all scanners - detect inter-scanner differences and vendor-dependent features. Analyse feature robustness to these sources of variability	N.A.	
Imaging at multiple time points - collect images of individuals at additional time points. Analyse feature robustness to temporal variabilities (for example, organ movement, organ expansion/shrinkage)	N.A.	For the retrospective nature of the study, it was not possible to acquire the same images at different time points.
Feature reduction or adjustment for multiple testing - decreases the risk of overfitting. Overfitting is inevitable if the number of features exceeds the number of samples. Consider feature robustness when selecting features	+3	Features reduction based on several feature selection techniques has been performed.
Multivariable analysis with non radiomics features (for example, EGFR mutation) - is expected to provide a more holistic model. Permits correlating/inferencing between radiomics and non radiomics features	N.A.	
Detect and discuss biological correlates - demonstration of phenotypic differences (possibly associated with underlying gene–protein expression patterns) deepens understanding of radiomics and biology	N.A.	
Cut-off analyses - determine risk groups by either the median, a previously published cut-off or report a continuous risk variable. Reduces the risk of reporting overly optimistic results	+1	
Discrimination statistics - report discrimination statistics (for example, C-statistic, ROC curve, AUC) and their statistical significance (for example, p-values, confidence intervals). One can also apply resampling method (for example, bootstrapping, cross-validation)	+2 (a discrimination statistic and its statistical significance are reported; a resampling method is also applied, i.e., cross-validation)	
Calibration statistics - report calibration statistics (for example, Calibration-in-the-large/slope, calibration plots) and their statistical significance (for example, P-values, confidence intervals). One can also apply resampling method (for example, bootstrapping, cross-validation)	N.A.	
Prospective study registered in a trial database - provides the highest level of evidence supporting the clinical validity and usefulness of the radiomics biomarker	N.A.	
Validation - the validation is performed without retraining and without adaptation of the cut-off value, provides crucial information with regard to credible clinical performance	+4 (Validation is based on two datasets from two distinct institutes)	
Comparison to 'gold standard' - assess the extent to which the model agrees with/is superior to the current 'gold standard' method (for example, TNM-staging for survival prediction). This comparison shows the added value of radiomics	N.A.	
Potential clinical utility - report on the current and potential application of the model in a clinical setting (for example, decision curve analysis).	N.A.	
Cost-effectiveness analysis - report on the cost-effectiveness of the clinical application (for example, QALYs generated)	N.A.	
Open science and data - make code and data publicly available. Open science facilitates knowledge transfer and reproducibility of the study	N.A.	
**RQS total score**	**+11**	

Table S7 - Radiomics Quality Score (RQS) checklist.
